# Curative effect of laparoscopic ureteral end-to-side anastomosis at the pelvic level for a duplex kidney in children

**DOI:** 10.3389/fped.2025.1509039

**Published:** 2025-02-19

**Authors:** Zedong Bian, Geng Xiong, Tong Liu, Yong Zhi, Ming Liu

**Affiliations:** Department of Pediatric Surgery, Affiliated Hospital of Southwest Medical University, Luzhou, Sichuan, China

**Keywords:** duplex kidney, laparoscopy, surgical procedure, ureteroureterostomy, children

## Abstract

**Background:**

To analyze the clinical efficacy and experience with laparoscopic ureteral end-to-side anastomosis at the pelvic level for a duplex kidney (DK) in children.

**Methods:**

This was a retrospective analysis of 20 children diagnosed with a complete DK in the pediatric surgery department of the Affiliated Hospital of Southwest Medical University between January 2018 and July 2024. The cohort comprised 15 girls and five boys aged 5–100 months (mean ± SD, 38.5 ± 29.9). There were 16 cases on the left side and four cases on the right side. There were five cases of simple upper ureterovesical junction stenosis, seven cases of upper ureteroceles, seven cases of upper ureteral ectopic opening, and one case of postoperative vesicoureteral reflux with fenestration for upper ureteroceles. The major clinical symptoms were intermittent perineal urinary leakage and repeated infection of the urinary tract. Laparoscopic duplex ureteral end-to-side anastomosis (between the end section of the upper ureter and the lateral section of the lower ureter) at the pelvic level was performed in all patients with an intraoperative indwelling ureteral stent.

**Results:**

Laparoscopy with no conversion to open was undertaken in the 20 cases. The duration of the procedure was 85–140 (112.9 ± 14.3) min. Intraoperative blood loss was 5–15 (8.2 ± 4.4) ml. Postoperative duration of hospital stay was 5–9 (6.5 ± 0.9) days. No anastomosis fistula, anastomotic stenosis, ureteral stump infection or other complications occurred. The ureteral stent was removed 8 weeks after surgery. All children were followed up for 4–68 (median, 32) months. Clinical symptoms disappeared, and the degree of upper renal hydronephrosis decreased.

**Conclusions:**

Laparoscopic ureteral end-to-side anastomosis at the pelvic level for a DK in children is safe and efficacious. It is a minimally invasive procedure that is simple with few complications, and merits wider popularization.

## 1 Introduction

A “duplex kidney” (DK) is a congenital condition in which one kidney divides into an upper part and a lower part; each part has its own independent renal pelvis and ureter. The worldwide prevalence of DK is ∼0.7% (1). A DK is significantly more common in girls than in boys. Usually, DK is combined with various types of pathologic morphology, such as upper ureterovesical junction stenosis (some children show upper ureteroceles), ectopic opening, and ureteral reflux. Previously, nephroureterectomy of the abnormal or diseased part of the DK and its connected ureter was the most commonly used surgical method for a DK. However, this surgical method has a relatively high prevalence of trauma and complications.

A surgical method of DK preservation has attracted attention. in 1928, Foley (2) first reported ureteroureterostomy as an alternative procedure for the treatment of a DK with obstructive lesions. With the development of minimally invasive methods, ureteroureterostomy has been applied in clinical practice gradually and shown to be a safe and efficacious surgical method (3, 4).

In this study, we present our initial data on laparoscopic duplex ureteral anastomosis at the pelvic level for children at our institution. The clinical efficacy and surgical experience are discussed herein.

## 2 Materials and methods

### 2.1 Patients

From January 2018 to July 2024, 20 children (15 girls and five boys; 16 cases on the left side and four cases on the right side) aged 5–100 months (mean ± SD, 38.5 ± 29.9) who underwent laparoscopic duplex ureteral end-to-side anastomosis at the pelvic level in the Department of Pediatric Surgery within the Affiliated Hospital of Southwest Medical University were analyzed retrospectively.

All patients underwent urinary ultrasound, computed tomography urography (CTU)/magnetic resonance urography and voiding cystourethrogram (VCUG) to confirm the diagnosis of a complete DK. There were five cases of simple upper ureterovesical junction stenosis, seven cases of upper ureteroceles, seven cases of upper ureteral ectopic opening, and one case of ureteral reflux after fenestration of upper ureteroceles. The main clinical symptoms were intermittent perineal leakage of urine and recurrent infection of the urinary tract. In the 20 patients in this study, both the upper and lower parts of the kidneys were functional. Fifteen of the patients had hydronephrosis; the degree of hydronephrosis was mild in two patients, moderate in eight, and severe in five.

The study inclusion criteria were: (1) the main clinical symptoms were urinary leakage and recurrent infection of the urinary tract; (2) ectopic or obstructive lesions of the distal upper ureter; (3) CTU showed a clear renal cortex and enhanced image in the upper kidney; (4) VCUG showed no vesicoureteral reflux.

The exclusion criteria were: (1) hydronephrosis of the lower DK; (2) vesicoureteral reflux detected by VCUG; (3) diuretic renogram showed no function of the upper kidney; (4) acute infection of the urinary tract.

### 2.2 Surgical methods

**Figure 1:**
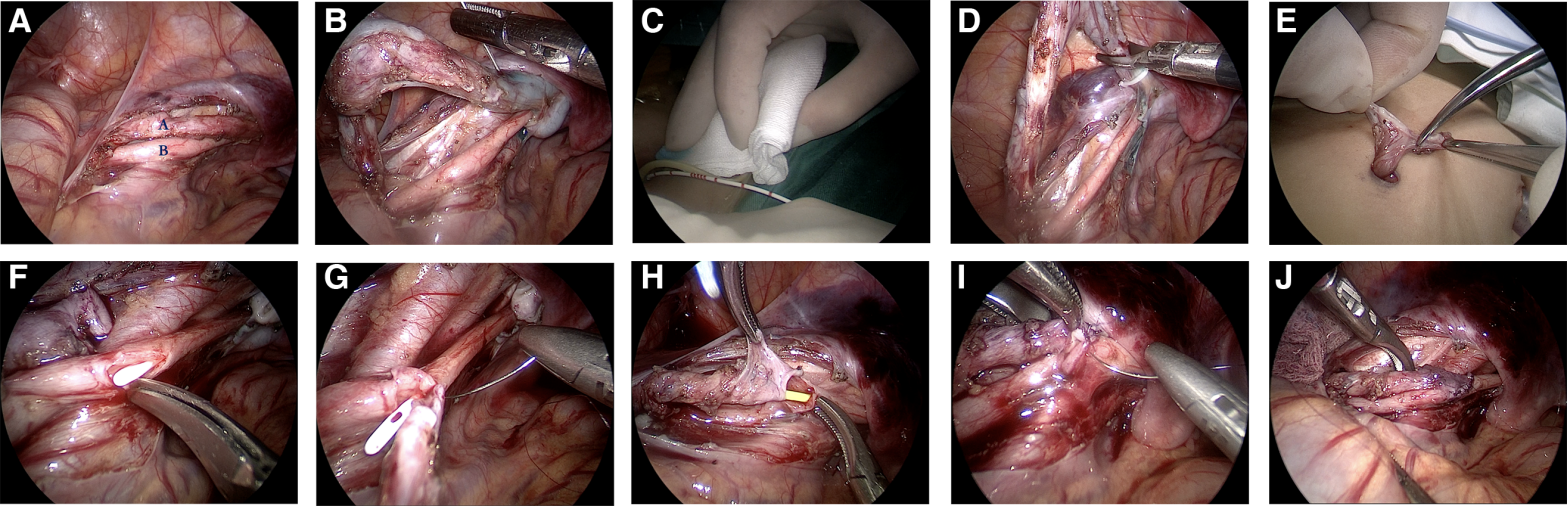
Images depicting the steps in total duplex ureteral end-to-side anastomosis at the pelvic level under laparoscopic guidance. **(A)** The duplex ureter is shown free at the level of the intersection of the ureter and iliac vessel on the affected side (**A**: upper ureter; **B**: lower ureter, which can be identified by the ureteral catheter in the lumen as an anatomical marker). **(B)** Methylene Blue was injected into the lumen of the upper ureter to ascertain if there was an ectopic opening during the procedure. **(C)** The gauze filled in the perineum was stained blue to confirm the ectopic opening of the upper ureter. **(D)** The upper ureter was severed by low pelvic ligation. **(E)** The proximal end of the upper ureter was pulled out through the skin incision of the trocar on the affected side and trimmed. The length of the ureteral opening was 1.5 cm. **(F)** The upper ureter was returned to the abdominal cavity, and the lower ureteral wall was cut 1.5 cm longitudinally on the level of the upper ureteral opening, and the pre-indwelling white ureteral catheter was visible in the lumen. **(G)** The anterior walls of the upper and lower ureter were sutured intermittently with a 6–0 absorbable suture. **(H)** Placement of the corresponding type of ureteral stent through the ureteral anastomosis. **(I)** The posterior wall of the upper and lower ureter was sutured intermittently with a 6–0 absorbable suture; **(J)** A Y-shaped ureter was formed without tension or torsion.

After tracheal intubation and general anesthesia, the child was placed in the lithotomy position. He/she underwent transurethral cystoscopy to ascertain the lower ureter of the affected side. An F3 or F4 ureteral catheter was placed retrograde through the opening of the lower ureter (used as an anatomic guidance marker to facilitate identification of the upper and lower ureters on the affected side during laparoscopic surgery). Then, the patient's position was changed to supine with the head down and feet up, and the patient (the whole body) tilted 30° to the healthy side. Pneumoperitoneum was established routinely, and the pressure of pneumoperitoneum was set at 6–12 mmHg (1 mmHg = 0.133 kPa). A 5 mm trocar was cut into the lower edge of the umbilicus, and a 3 mm trocar or a 5 mm trocar was placed at two points: at the flat umbilicus with the outer edge of the abdominus rectus muscle on the affected side, and the midpoint between the anterior superior iliac spine and the umbilicus on the healthy side. A longitudinal incision of the pelvic peritoneum was made at the level of the intersection between the ureter and iliac blood vessel on the affected side using an electric coagulation hook. For boys, the incision of the peritoneum was made within the spermatic-cord blood vessel and above the vas deferens on the affected side. For girls, the incision of the peritoneum was made inside within ovarian vessels and above the ovary on the affected side. The upper and lower ureters were dissociated and fully exposed (Figure 1A). Methylene Blue solution was injected into the upper ureter (Figure 1B), and the perineal sterile gauze was stained blue (Figure 1C), which reconfirmed the upper ureter as a duplex ureter with an ectopic opening. The upper ureter was dissociated to the lower pelvic cavity, and the upper ureter was ligated and severed (Figure 1D). The proximal end of the upper ureter was pulled out through the skin incision of the trocar on the affected side. The length of the ureteral opening was trimmed to ∼1.5 cm (if the diameter of the ureter was >1.5 cm, then the ureter had to be cut to form appropriately) (Figure 1E). The trimmed upper ureter was returned to the abdominal cavity and placed in a tension-free position. The lower ureteral wall was incised longitudinally by ∼1.5 cm (Figure 1F). The anterior walls of the upper and lower ureter were sutured intermittently with 6–0 absorbable sutures (Figure 1G). A suitable type of ureteral stent was inserted through the anastomosis (the distal end of the ureteral stent was inserted into the lower ureter and bladder, while the proximal end was placed into the upper ureter and upper renal pelvis). The proximal end was placed in the upper ureter above the anastomosis, and the distal end was placed in the bladder (Figure 1H). The posterior walls of the upper and lower ureter were interrupted with a 6–0 absorbable suture (Figure 1I). A Y-shaped ureter was formed without tension or torsion (Figure 1J).

### 2.3 Statistical analyses

SPSS 22.0 (IBM, Armonk, NY, USA) was used for statistical analyses. Measurement data are expressed as the mean ± standard deviation. Counting data are expressed as percentages.

## 3 Results

All 20 patients underwent laparoscopic duplex ureteral end-to-side anastomosis at the pelvic level without the need for conversion to open surgery. The operative time was 85–140 (mean ± SD, 112.9 ± 14.3) min. Blood loss was 5–15 (mean ± SD, 8.2 ± 4.4) ml. The postoperative duration of hospital stay was 5–9 (mean ± SD, 6.5 ± 0.9) days. Postoperative complications such as anastomotic leakage, anastomotic stenosis, or infection of the ureteral distal stump were not observed.

The ureteral stent was removed 8 weeks after surgery. Patients were followed up for 4–68 (median, 32) months. The symptom of urinary leakage disappeared completely in all patients, and no infection of the urinary tract recurred, as shown in Table 1.

**Table 1:** Relevant data for the cohort (*N* = 20).

Number of cases (male/female)	20 (5/15)
Age (months), mean ± SD (range)	38.5 ± 29.9 (5–100)
Classification of disease	
Simple duplex ureteral terminal stricture, *n* (%)	5 (25.0)
Duplex ureteral terminal cyst, *n* (%)	7 (35.0)
Ectopic ureter of duplex ureter, *n* (%)	7 (35.0)
Ureteral reflux after fenestration of duplex ureteral cysts, *n* (%)	1 (5.0)
Duration of procedure (min), mean ± SD (range)	112.9 ± 14.3 (85–140)
Intraoperative blood loss (ml), mean ± SD (range)	8.2 ± 4.4 (5–15)
Postoperative duration of hospital stay (d), mean ± SD (range)	6.5 ± 0.9 (5–9)
Postoperative complications	
Anastomotic leak, *n* (%)	0 (0)
Anastomotic stenosis, *n* (%)	0 (0)
Infection of ureteral stump, *n* (%)	0 (0)
Postoperative follow-up	
Period (months), median (range)	32 (4–68)
Leakage of urine, *n* (%)	0 (0)
Infection of the urinary tract, *n* (%)	0 (0)

## 4 Discussion

A DK is a common malformation of the upper urinary tract in pediatric urology. A DK and ureter malformation are usually divided into “complete” and “incomplete” types.

In children with a DK, the upper kidney is usually associated with distal ureteral obstructive lesions (including simple ureterovesical junction stenosis and ureteroceles) or ureteral ectopic opening; the lower kidney is often associated with vesicoureteral reflux (5, 6). The clinical manifestations of a DK vary between individuals. If clinical symptoms and signs are absent, then a DK does not need treatment. If it is combined with ureteral ectopic opening, hydronephrosis, and progressive aggravation of ureteral dilatation, vesicoureteral reflux or ureteroceles, it may cause persistent leakage of urine or recurrent infection of the urinary tract, which often necessitate surgical intervention (7, 8).

There are various surgical methods for the treatment of a DK malformation (9). Heminephrectomy (10) is often used in the early stage, but the blood supply to the lower kidney and lower ureter may be damaged by the separation and traction of the renal-hilum tissue during upper nephrectomy, which is traumatic. Multicenter studies (11) have shown that the risk of lower-kidney injury during heminephrectomy in children is 5%–9%. For children with clinical symptoms and upper-kidney function, DK-sparing surgery can be adopted according to the specific situation. Common surgical methods include ureteral side-to-side anastomosis and ureterovesical reimplantation (12, 13).

With the development of minimally invasive technology, duplex ureteral end-to-side anastomosis has been applied gradually in clinical practice, and has been shown to be a safe and efficacious surgical method (14–16). Its main advantage is that it is minimally invasive. Only uretero–ureteral anastomosis is undertaken, which avoids the risk of vascular injury caused by the procedure to the renal hilum, and retains the function of the DK to a maximum extent, and the risk is significantly lower than that of heminephrectomy (17). It has been reported (8, 18) that ureteroureterostomy is safe and efficacious for children with renal function <10% or no function in the DK.

Duplex end-to-side ureteroureterostomy can be anastomosed with the proximal ureter at the lower pole of the kidney or with the distal ureter at the pelvic level. The choice of which level should be based on the experience and habits of the surgeon (14). Ureteroureterostomy at the lower pole of the kidney is suitable for patients with extremely severe dilatation and obvious tortuosity of the upper ureter. If a low anastomosis is carried out, then urine may not drain well after surgery. Therefore, the upper ureter can be cut off at the relatively normal proximal end, and the upper ureteral opening can be cut and then anastomosed with the lower ureter. However, the disadvantage of this surgical method is that if the anastomosed stenosis after surgery requires reoperation, it may face the lack of length with the upper ureter and the difficulty of location selection, which makes the ureter reanastomosis extremely difficult. We believe that for patients with mild-to-moderate ureteral dilatation and no obvious tortuosity of the upper ureter in a DK, the end-to-side pelvic ureteral anastomosis is more reasonable because it has seven main advantages.

First, at the pelvic level, the anatomical position near the intersection of the ureter and iliac vessels is shallow. It is easier to locate the upper and lower ureter when the patient's position is supine with the head down and feet up, and the body is tilted to the healthy side; in this position, the ureter is quick and convenient to free. Second, in this area, except for the iliac vessels, there are few important organs, the free range of the colon is small, the gonad vessels are clearly exposed, the anatomical range is small, and the probability of causing injury to adjacent tissue is low. Third, there is no need for large-scale mobilization of the upper ureter, which minimizes the risk of lower-ureter injury and fully protects its blood supply. Fourth, the procedure at the pelvic level can ensure that the distal end of the upper ureter can be cut as low as possible in the pelvic cavity to avoid infection of the ureteral distal stump. Fifth, there are fewer important organs around the ureter in the pelvic level, and the operative space is large, which is conducive to duplex ureteral end-to-side anastomosis. Sixth, if choosing the pelvic level for ureteral anastomosis, there is no need to remove most of the upper ureter. If the anastomotic stenosis requires reoperation after surgery, it is easy to release the local adhesion, and there is sufficient material for the upper ureter to be free for ureteral anastomosis or ureteral reimplantation. Seventh, duplex ureteral anastomosis at the pelvic level may also reveal problems at the distal end of the lower ureter, so they can be treated together intraoperatively.

The common complications after duplex ureteral end-to-side anastomosis are anastomotic leakage, anastomotic stenosis, and infection of the ureteral distal stump. Gerwinn et al. (9) reported two cases (12.5%) of anastomotic leakage after laparoscopic ureteral anastomosis. The clinical manifestations were febrile infection of the urinary tract and paralytic ileus, and they were cured by conservative treatment. Mcleod et al. (15) reported 43 children with a DK treated by this procedure, and the prevalence of anastomotic stenosis was 2%. Smith et al. (19) found that in children with a DK treated with ureteral end-to-side anastomosis, the prevalence of infection of the ureteral distal stump was low, with a reoperation prevalence of 6.7%.

In 20 children with a DK, we chose duplex laparoscopic ureteral end-to-side anastomosis at the pelvic level. There were no postoperative complications such as anastomotic leakage, anastomotic stenosis, or infection of the ureteral distal stump. The surgeons in our team have rich experience in laparoscopic reconstruction of the upper urinary tract in children and robust technology for laparoscopic anastomosis. They are familiar with the anatomical structure of the surgical area, which is the prerequisite for ensuring postoperative recovery. Irrespective of the surgical level chosen, the key factors affecting the success of the procedure are: careful identification of the double ureters intraoperatively; avoiding damage to the blood supply of the ureter as much as possible; freeing the ureter to the distal end and ligating the upper ureter at a low position as much as possible; selecting the appropriate anastomosis; ensuring that the anastomosis is free of tension and torsion.

We documented six major experiences for laparoscopic duplex ureteral end-to-side anastomosis at the pelvic level. First, before laparoscopic surgery, a ureteral catheter of the corresponding model was placed retrograde into the lower ureter under cystoscope guidance, which was used as an anatomical marker to facilitate accurate identification of the upper and lower ureter during the procedure and to avoid accidental injury. Moreover, due to the support of the ureteral catheter, the lower ureteral lumen could maintain a certain tension, which was convenient for the surgeon to cut the lower ureteral wall longitudinally. Simultaneously, it could prevent the lower ureter from being accidentally cut off completely during the incision. Second, the duplex upper and lower ureters are, in general, in the same sheath, so special attention should be paid to protecting the blood supply of the ureter when releasing the ureter. We recommend that a 3-mm electric coagulation hook and pointed scissors should be used (if possible) to ensure accurate cutting, protect the normal blood supply of the ureter as much as possible, and also facilitate the longitudinal incision of the ureteral wall. The dissociation range of the co-sheath ureter should not be too long, and it can achieve low transection of the lower ureter and ensure a tension-free anastomosis. Third, before longitudinal incision of the lower ureter, the obstructed dilated upper ureter can be dissected first because the dilated upper ureter will shrink to a certain extent after decompressing, and the diameter will also be reduced, which is convenient to accurately select the position and size of the ureteral anastomosis in the tension-free state. Fourth, we believe that the severed upper ureter can be pulled out of the body through the skin incision of the trocar for repair. This strategy can help to fix the ureter and facilitate the procedure, ensure accurate cutting of the ureteral opening, shorten the duration of the procedure, avoid repeated clamping of the ureter, protect the normal blood supply of the ureter, and ensure the suitability of the ureteral opening anastomosis. Fifth, intraoperatively, the lower ureter should be dissociated as little as possible, and clamping the ureter should be avoided as much as possible to avoid affecting its normal blood supply and leading to anastomotic stenosis. The ureter at the pelvic entrance is relatively fixed, so it can be separated, cut, and anastomosed readily. If necessary, the lower ureter can be suspended from the rear through the abdominal-wall indwelling traction line to avoid puncture of the ureteral wall and reduce the probability of ureteral injury/stricture. Sixth, on the premise of tension-free anastomosis, the position of the lower-ureter incision should be selected at the point where the ureter enters the pelvis because this position of the ureter is the most superficial, the easiest to expose, and the difficulty of anastomosis is low. If the incision position is too low, it will increase the difficulty of ureteral mobilization and anastomosis.

Our study had two main limitations. The study cohort was small and the follow-up time was short.

## 5 Conclusions

Laparoscopic duplex ureteral end-to-side anastomosis at the pelvic level is safe and efficacious for the treatment of a DK in children. It is a simple procedure associated with little trauma and few complications. This procedure is worthy of promotion.

## Data Availability

The raw data supporting the conclusions of this article will be made available by the authors, without undue reservation.
